# Rapid decline in kidney function is associated with rapid deterioration of health-related quality of life in chronic kidney disease

**DOI:** 10.1038/s41598-023-28150-w

**Published:** 2023-01-31

**Authors:** Hyo Jin Kim, Da Woon Kim, Harin Rhee, Sang Heon Song, Sue Kyung Park, Soo Wan Kim, Yeong Hoon Kim, Suah Sung, Kook-Hwan Oh, Eun Young Seong

**Affiliations:** 1 Department of Internal Medicine, Pusan National University Hospital, Pusan National University College of Medicine, Busan, South Korea; 2 Biomedical Research Institute, Pusan National University Hospital, Busan, South Korea; 3 Department of Preventive Medicine, Seoul National University College of Medicine, Seoul, South Korea; 4 Department of Internal Medicine, Chonnam National University Medical School, Gwangju, South Korea; 5 Department of Internal Medicine, Inje University Busan Paik Hospital, Busan, South Korea; 6 Department of Internal Medicine, Eulji Medical Center, Eulji University, Seoul, South Korea; 7 Department of Internal Medicine, Seoul National University Hospital, Seoul National University College of Medicine, Seoul, South Korea

**Keywords:** Nephrology, Kidney diseases

## Abstract

This study aimed to evaluate changes in health-related quality of life (HRQOL) in patients with chronic kidney disease (CKD) according to decline in kidney function. HRQOL was assessed using the Short Form-36 questionnaire composed of a physical component summary (PCS) and mental component summary (MCS). Rapid decline in kidney function was defined as a decline in the estimated glomerular filtration rate (eGFR) of > 3 mL/min/1.73 m^2^/year. Rapid deterioration of HRQOL was defined a change in the HRQOL value greater than the median. Among 970 patients, 360 (37.1%) were in the rapid kidney function decline group. In 720 patients who were 1:1 propensity score-matched, the baseline eGFR was not significantly different between the non-rapid and rapid kidney function decline groups. Compared with the baseline PCS score, the 5-year PCS score decreased in the non-rapid and rapid kidney function decline groups. The 5-year MCS score significantly decreased in the rapid kidney function decline group alone. Rapid decline in kidney function was significantly associated with rapid deterioration of the PCS (odds ratio [OR]: 1.48; 95% confidence interval [CI]: 1.07–2.05; *P* = 0.018) and MCS (OR: 1.89; 95% CI 1.36–2.62; *P* < 0.001) scores. Rapid decline in kidney function was associated with rapid deterioration of HRQOL in patients with CKD.

## Introduction

Quality of life (QOL) is the degree of well-being or happiness at which an individual is comfortable, healthy, and able to participate in or enjoy life events. Health-related quality of life (HRQOL) represents a patient’s comprehensive perception of mental and physical health and is regarded as a health outcome. Renal and extrarenal conditions can lead to poor HRQOL in patients with chronic kidney disease (CKD). Patients with advanced CKD have various complex comorbid conditions that can lead to poor HRQOL^1^. HRQOL scores are significantly lower in patients with CKD of all stages, including dialysis, than in the general population^2^. According to the results of the KoreaN Cohort Study for Outcome in Patients With Chronic Kidney Disease (KNOW-CKD), which comprised a South Korean CKD cohort, the HRQOL scores are lower in patients with advanced CKD compared to early CKD^3^.

Low HRQOL is associated with poor patient outcomes. A previous study performed on African Americans with hypertensive CKD showed that low physical or mental HRQOL is associated with an increased risk of cardiovascular events/cardiovascular death and CKD progression or death^4^. Moreover, cardiovascular outcomes and mortality are associated with low HRQOL in patients with predialysis CKD^5,6^. As CKD progresses, HRQOL can decrease, which may result in poor patient outcomes; therefore, monitoring HRQOL is important.

In patients with CKD, kidney function declines over time; some patients have a rapid decline in kidney function, while others do not. Lack of energy, drowsiness, and fatigue are common symptoms that result in poor HRQOL in patients with predialysis CKD^7^. HRQOL could decrease according to kidney function decline; however, little is known about how a rapid decline in kidney function affects HRQOL changes. Therefore, this study aimed to evaluate changes in HRQOL in patients with CKD according to rapid kidney function decline using data obtained from a large Korean CKD cohort.

## Results

### Participants’ characteristics

**Figure 1:**
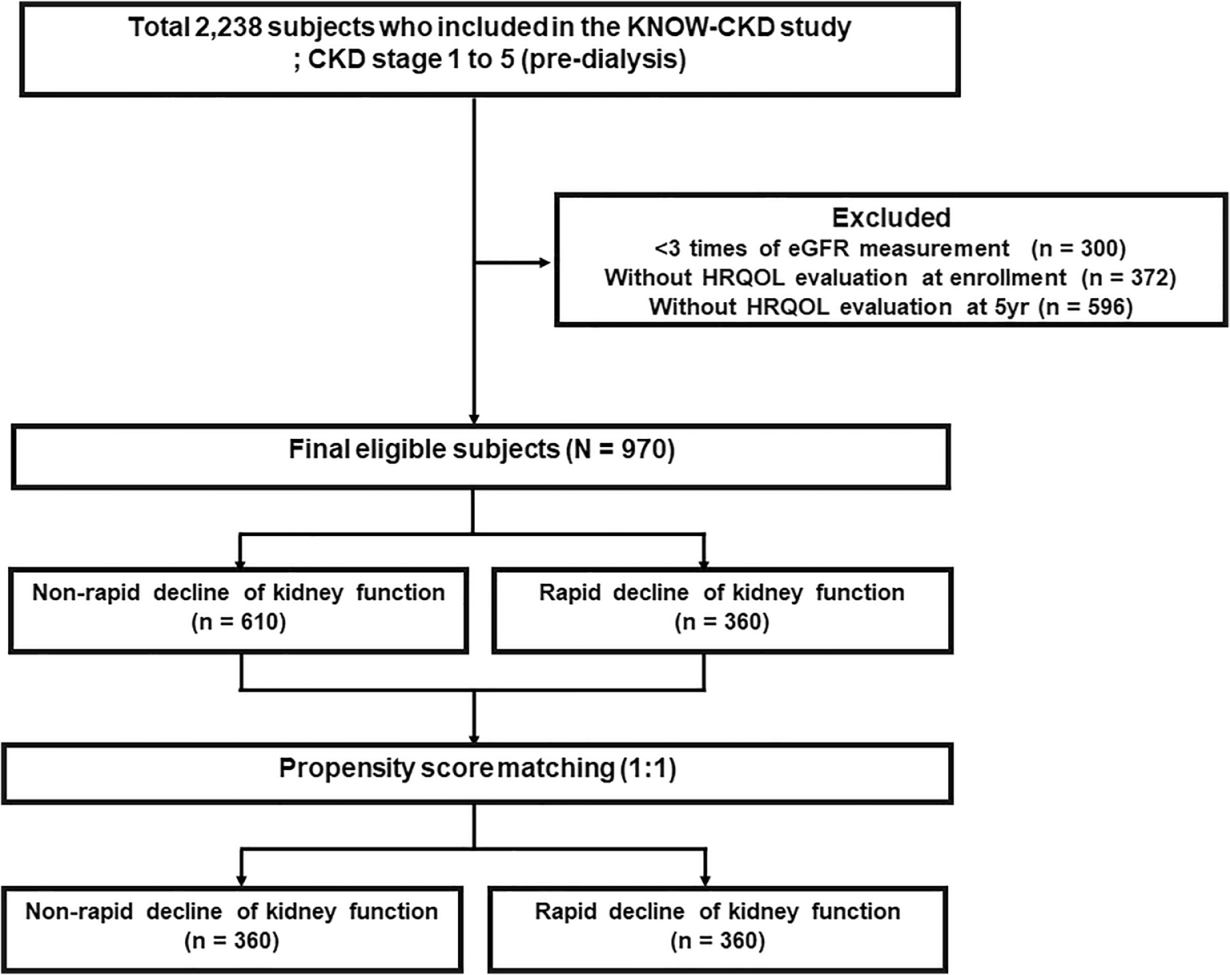
Flow chart of patient enrollment and analyses. CKD, chronic kidney disease; eGFR, estimated glomerular filtration rate; HRQOL, health-related quality of life.

In 2,238 patients enrolled in the KNOW-CKD cohort between 2011 and 2016, 970 participants had an estimated glomerular filtration rate (eGFR) of more than three times during the follow-up period and underwent HRQOL evaluation at enrollment and at 5 years (Fig. 1). The clinical characteristics of the study patients, stratified by the presence of a rapid decline in kidney function, are shown in Table 1. The mean age was 51.9 ± 12.1 years, and 62.3% of the subjects were male. The mean eGFR was 57.6 ± 30.4 mL/min/1.73 m^2^. Among all patients, 37.1% (360 patients) were in the rapid decline group. Patients with diabetes mellitus (DM) (*P* = 0.017) and hypertension (HTN) (*P* = 0.007) were more likely to experience a rapid decline in kidney function. The baseline eGFR value (*P* < 0.001) was lower in the rapid kidney function decline group.

**Table 1:** Baseline clinical characteristics of patients, stratified according to the presence of a rapid decline in kidney function.

Characteristics	Before PSM				After PSM			
	Total (N = 970)	Non-rapid decline group (n = 610)	Rapid decline group (n = 360)	*P* value	Total (N = 720)	Non-rapid decline group (n = 360)	Rapid decline group (n = 360)	*P* value
Age (mean ± SD)	51.9 ± 12.1	52.5 ± 12.3	50.9 ± 11.8	0.045	51.5 ± 11.7	52.1 ± 11.6	50.9 ± 11.8	0.174
Sex, male, n (%)	604 (62.3)	368 (60.3)	236 (65.6)	0.105	468 (65.0)	232 (64.4)	236 (65.6)	0.755
BMI (kg/m^2^)	24.5 ± 3.4	24.3 ± 3.3	24.8 ± 3.5	0.017	24.6 ± 3.4	24.4 ± 3.4	24.8 ± 3.5	0.104
MBP (mmHg)	93.3 ± 10.5	92.6 ± 10.5	94.4 ± 10.5	0.008	93.6 ± 10.4	92.7 ± 10.3	94.4 ± 10.5	0.025
DM, n (%)	261 (27.0)	148 (24.3)	113 (31.4)	0.017	207 (28.8)	94 (26.1)	113 (31.4)	0.118
HTN, n (%)	933 (96.2)	579 (94.9)	354 (98.3)	0.007	702 (97.5)	348 (96.7)	354 (98.3)	0.152
Smoking status, n (%)				0.325				0.941
Never	513 (52.9)	330 (54.1)	183 (50.8)		367 (51.0)	353 (49.0)	183 (50.8)	
Current or former	457 (47.1)	280 (45.9)	177 (49.2)		353 (49.0)	176 (48.9)	177 (49.2)	
Low income*, n (%)	172 (18.2)	102 (17.2)	70 (19.6)	0.351	133 (18.7)	63 (17.8)	70 (19.6)	0.217
Low education**, n (%)	451 (46.5)	267 (43.8)	184 (51.1)	0.027	388 (46.9)	154 (42.8)	184 (51.1)	0.025
Employed, n (%)	614 (63.3)	378 (62.0)	236 (65.6)	0.263	457 (63.5)	221 (61.4)	236 (65.6)	0.246
eGFR (mL/min/1.73m^2^)	57.6 ± 30.4	62.1 ± 31.6	49.9 ± 26.7	< 0.001	49.7 ± 26.7	49.5 ± 26.7	49.9 ± 26.7	0.845
eGFR slope (ml/min/1.73m^2^/year)	- 3.54 (- 2.25, - 1.05)	- 1.39 (- 2.12, - 0.48)	- 4.01 (- 5.11, - 3.40)	< 0.001	- 3.00 (- 4.01, - 1.51)	- 1.52 (- 2.24, - 0.49)	- 4.01 (- 5.11, - 3.40)	< 0.001
Hemoglobin (g/dL)	13.1 ± 1.9	13.4 ± 1.9	12.7 ± 1.9	< 0.001	12.9 ± 2.0	13.2 ± 2.0	12.7 ± 1.9	0.003
Uric acid (mg/dL)	7.0 ± 1.9	6.8 ± 1.9	7.3 ± 1.8	0.001	7.3 ± 1.9	7.3 ± 1.8	7.3 ± 1.8	0.945
Albumin (g/dL)	4.2 ± 0.4	4.3 ± 0.4	4.2 ± 0.4	< 0.001	4.2 ± 0.4	4.3 ± 0.4	4.2 ± 0.4	0.001
Total cholesterol (mg/dL)	174.2 ± 37.3	173.5 ± 35.7	175.4 ± 39.9	0.440	173.0 ± 37.8	170.6 ± 35.5	175.4 ± 39.9	0.090
UPCR, median, (Q1, Q3) (g/g)	0.6 (0.2, 1.5)	0.6 (0.2, 1.5)	0.6 (0.2, 1.6)	0.889	0.6 (0.2, 1.6)	0.6 (0.3, 1.6)	0.6 (0.2, 1.6)	0.639
UPCR, median (Q1, Q3) (g/g)	0.4 (0.1, 1.2)	0.3 (0.1, 0.7)	0.7 (0.2, 2.1)	< 0.001	0.5 (0.2, 1.4)	0.4 (0.1, 0.9)	0.7 (0.2, 2.1)	< 0.001
PCS	75.3 ± 16.4	76.1 ± 16.2	73.8 ± 16.7	0.034	74.6 ± 16.8	75.3 ± 16.9	73.8 ± 16.7	0.242
MCS	71.6 ± 17.0	72.2 ± 17.1	70.7 ± 16.8	0.193	71.1 ± 17.3	71.4 ± 17.8	70.7 ± 16.8	0.578

The rapid decline in kidney function was defined as a decline in eGFR of > 3 mL/min/1.73 m^2^/year.

*Low-income status was defined as a monthly family income of less than ₩1,500,000 (approximately 1500 US dollars).

**Low education level was defined as an academic background less than high school graduation because high school education is compulsory in Korea.

PSM, propensity score matching; SD, standard deviation; BMI, body mass index; MBP, mean blood pressure; DM, diabetes mellitus; HTN, hypertension; eGFR, estimated glomerular filtration rate, as determined by the CKD-EPI, creatinine equation; CRP, C-reactive protein; Ca, calcium; UPCR, urine protein-to-creatinine ratio; PCS, physical component summary; MCS, mental component summary.

As there was a significant difference in baseline kidney function between the two groups, we performed propensity score matching (PSM) to minimize this difference. In propensity score-matched patients, the baseline eGFR was not significantly different between the non-rapid and rapid kidney function decline groups (non-rapid vs. rapid decline groups: 49.5 ± 26.7 vs. 49.9 ± 26.7 mL/min/1.73 m^2^; *P* = 0.845). In the propensity score-matched patients, the mean age (*P* = 0.174) and body mass index (BMI) (*P* = 0.104) were similar between the non-rapid and rapid kidney function decline groups. The number of patients with DM (*P* = 0.118) and HTN (*P* = 0.0.152) was also similar in both the groups. The mean eGFR slope was −4.01 (−5.11, −3.40) mL/min/1.73 m^2^/year in the rapid kidney function decline group, whereas it was −1.52 (−2.24, −0.49) mL/min/1.73 m^2^/year in the non-rapid kidney function group. In the baseline HRQOL values of propensity score-matched patients, there was no significant difference in the physical component summary (PCS; *P* = 0.242) or mental component summary (MCS; *P* = 0.578) score between the non-rapid and rapid kidney function decline groups.

### HROQL changes during the follow-up period

**Figure 2:**
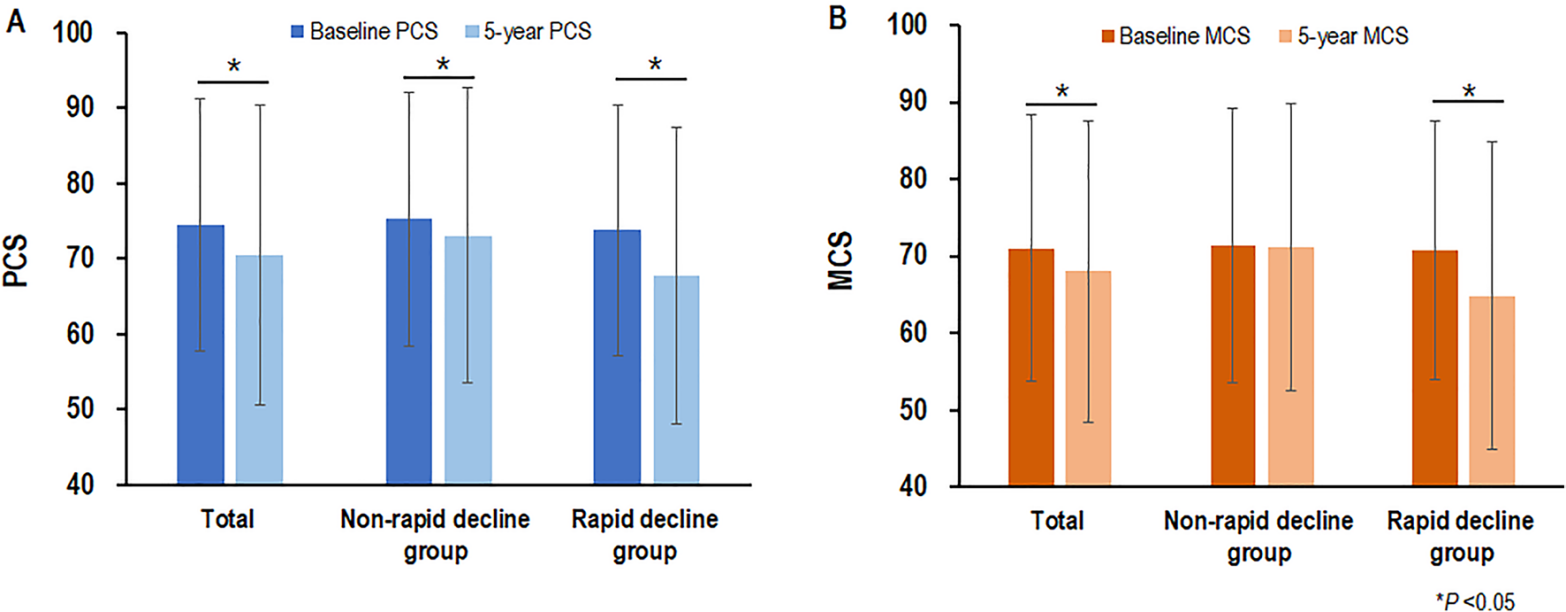
HRQOL changes according to the presence of a rapid decline in kidney function after propensity score matching. (**A**). The PCS score was significantly decreased in both the non-rapid (*P* = 0.012) and rapid (*P* < 0.001) kidney function decline groups. (**B**). The MCS score was significantly decreased in the rapid kidney function decline group alone (*P* < 0.001). HRQOL, health-related quality of life; PCS, physical component summary; MCS, mental component summary.

The baseline PCS and MCS scores were 74.6 ± 16.8 and 71.1 ± 17.3, respectively. Among the follow-up surveys, both the 5-year-PCS (70.4 ± 19.9; *P* = 0.001) and 5-year-MCS (68.0 ± 19.5; *P* < 0.001) scores were significantly decreased compared with their respective baseline scores (Fig. 2). According to the rapid decline in kidney function, the PCS score significantly decreased in the non-rapid (*P* = 0.012) and rapid (*P* < 0.001) kidney function decline groups (Fig. 2A). The MCS score significantly decreased in the rapid kidney function decline group alone (*P* < 0.001; Fig. 2B).

**Figure 3:**
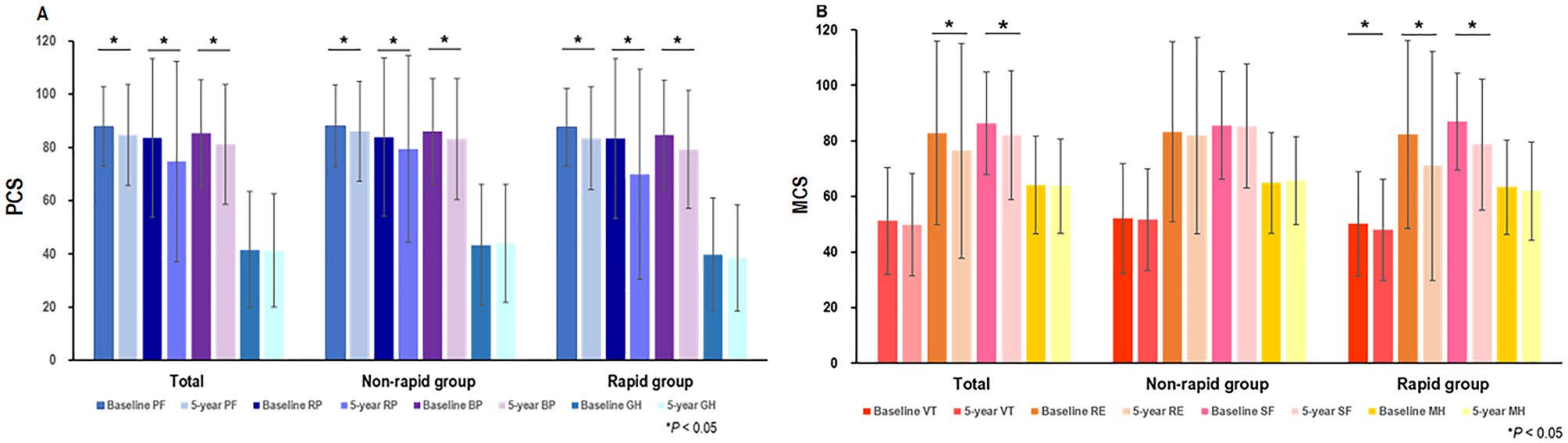
PCS and MCS component changes according to the presence of a rapid decline in kidney function. (**A**) Changes in PCS component scores. The scores for physical function, role-physical, and bodily pain decreased significantly at 5 years in the entire group, as well as in the non-rapid and rapid kidney function decline groups. (**B**) Changes in MCS component scores. The scores for role-emotional and social function decreased at the 5-year follow-up. In the rapid kidney function decline group, those for vitality, role-emotional, and social function significantly decreased at the 5-year follow-up. However, no significant decrease in the MCS score was observed in the non-rapid kidney function decline group during the follow-up period. PCS, physical component summary; PF, physical function; RP, role-physical; BP, bodily pain; GH, general health; MCS, mental component summary; VT, vitality; RE, role-emotional; SF, social function; MH, mental health.

The scores for physical function, role-physical, and bodily pain (i.e., PCS components) decreased significantly at 5 years in the entire patient cohort, as well as in the non-rapid and rapid kidney function decline groups (Fig. 3A). The scores for role-emotional and social function (i.e., MCS components) decreased at the 5-year follow-up. In the rapid kidney function decline group, the scores for vitality, role-emotional, and social function significantly decreased at the 5-year follow-up (Fig. 3B). However, no significant decrease in the MCS score was observed in the non-rapid kidney function decline group during the follow-up period (Fig. 3B).

### Association between kidney function decline and HRQOL deterioration

In an unadjusted binary logistic regression model, rapid kidney function decline was a significant factor related to rapid HRQOL deterioration in terms of both the PCS (odds ratio [OR]: 1.55; 95% confidence interval [CI]: 1.15–2.07; *P* = 0.004) and MCS (OR:1.79; 95% CI 1.33–2.40; *P* < 0.001) scores (Table 2). This association remained consistent after full adjustment for confounding variables, which included age, sex, HTN, DM, mean blood pressure, BMI, education level, income level, employment status, hemoglobin, uric acid, albumin, and total cholesterol levels, baseline eGFR, and log urine protein-to-creatinine ratio. Rapid kidney function decline was significantly associated with rapid deterioration of the PCS (OR: 1.48; 95% CI 1.07–2.05; *P* = 0.018) and MCS (OR: 1.89; 95% CI 1.36–2.62; *P* < 0.001) scores in model 3. In addition, rapid HRQOL deterioration was not significantly associated with the CKD causes (diabetic nephropathy, hypertensive nephropathy, glomerulonephritis, polycystic kidney disease, and others) and underlying cardiovascular disease. Using angiotensin-converting enzyme inhibitors or angiotensin receptor blockers, and calcium-based phosphate binders did not affect the rapid deterioration in HRQOL.

**Table 2:** Odds ratio of HRQOL decline according to the rapid decline in kidney function.

	Unadjusted		Model 1		Model 2		Model 3	
	OR (95% CI)	*P* value	OR (95% CI)	*P*-value	OR (95% CI)	*P *value	OR (95% CI)	*P* value
After PSM								
PCS								
Rapid decline of kidney function	1.55 (1.15, 2.07)	0.004	1.52 (1.13. 2.05)	0.006	1.50 (1.11, 2.02)	0.009	1.48 (1.07, 2.05)	0.018
MCS								
Rapid decline of kidney function	1.79 (1.33, 2.40)	< 0.001	1.78 (1.32, 2.41)	< 0.001	1.81 (1.34, 2.45)	< 0.001	1.89 (1.36, 2.62)	< 0.001

The rapid decline in kidney function was defined as a decline in eGFR of > 3 mL/min/1.73 m^2^/year.

Model 1: Adjusted for age, sex, HTN, DM, mean blood pressure, and BMI.

Model 2: Model 1 + education, income, and employment.

Model 3: Model 2 + hemoglobin, uric acid, albumin, total cholesterol, baseline eGFR, and logUPCR.

PSM, propensity score matching; OR, odds ratio; CI, confidence interval; DM, diabetes mellitus; HTN, hypertension; BMI, body mass index; eGFR, estimated glomerular filtration rate by CKD-EPI creatinine equation; UPCR, urine protein-to-creatinine ratio.

### Subgroup analyses in propensity score-matched patients

The association between rapid kidney function decline and HRQOL deterioration, categorized according to age, sex, presence of DM, and eGFR, is shown in Fig. S1. The rapid decline in kidney function was a significant risk factor for the rapid deterioration of the PCS score in patients with an eGFR of < 45 mL/min/1.73 m^2^, those with an age of < 65 years, or male individuals (Fig. S1A). The rapid decline in kidney function was a significant risk factor for the rapid deterioration of the MCS score in patients with an eGFR of ≥ 45 or < 45 mL/min/1.73 m^2^, those with an age of < 65 years, male individuals, or patients without diabetes (Fig. S1B).

## Discussion

In this study, the PCS and MCS scores decreased significantly during the follow-up period. The PCS score decreased significantly in both the non-rapid and rapid kidney function decline groups, and the MCS score decreased significantly in the rapid kidney function decline group alone. Even in propensity score-matched patients with similar basal renal function in the non-rapid and rapid kidney function decline groups, the rapid decline in kidney function was a significant risk factor for the rapid deterioration of the PCS and MCS scores.

In this study, patients in both the non-rapid and rapid kidney function decline groups showed a decrease in the PCS score during the follow-up period. In addition, the PCS component scores, including those for physical function, role-physical, and bodily pain, significantly decreased. According to national surveys in France, patients with CKD had lower HRQOL than the general population (in terms of symptoms, burden, and effects of kidney disease)^2^. Symptoms related to CKD often occur in clusters, and this symptom burden is a predictor of HRQOL decline^8,9^. In patients with CKD, especially advanced CKD, symptoms such as lack of energy, drowsiness, fatigue, and pain are prominent^7^. This symptom burden can lead to negative physical, psychological, and emotional responses^10^. Therefore, when treating patients with CKD, the full range of symptoms should be assessed and applied to improve their QOL and the quality of care.

General health, which represents overall general health awareness, did not decrease significantly during the follow-up period in this study. However, the general health score was much lower at baseline than those for the other PCS components. The Heart and Soul Study showed that self-assessed overall health was more reduced in earlier stages of renal function than mental health outcomes or QOL^11^. Another study also showed that HRQOL dimensions—physical health and bodily pain—remain almost stable over the course of CKD^12^. However, the perception of general health presents a declining trend to an important extent. Therefore, it is necessary to pay attention to patients’ health awareness and to educate them on improving their HRQOL. Since a patient's symptom burden can reduce their HRQOL, clarifying these symptoms and approaching treatment will help improve HRQOL. HRQOL is one of the many patient-centered outcomes that can be assessed using patient-reported outcome measures. Incorporating patient-reported outcomes into clinical care is crucial for providing patient-centered care and improving the patients’ QOL^13^. Therefore, efforts are being made to focus on patient-reported results in patients with CKD, and studies in which symptom burden was relieved have also been reported^14,15^.

The MCS score was significantly decreased in the rapid kidney function decline group but not in the non-rapid kidney function decline group. The MCS component scores also significantly decreased in the rapid kidney function decline group alone. That is, when patients did not have rapidly decreasing kidney function, the PCS score decreased, although the MCS score did not decrease significantly. This suggests that the mental health composition of patients with CKD was better tolerated in the absence of rapid kidney function decline. Although not evaluated in patients with rapid decline in kidney function, a previous study showed that CKD stage 2-3b had no significant effect on mental HRQOL^16^. Another study showed that physical health is affected in patients with moderate CKD; however, mental health outcomes remain stable^11^. Therefore, physicians should be concerned with and supportive of patients’ mental health.

In advanced CKD (eGFR < 45 mL/min/1.73 m^2^), rapid decline in kidney function was a significant risk factor for rapid deterioration of both the PCS and MCS scores. This suggests that not only is HRQOL lower in advanced CKD, but the rate of deterioration is also faster. Although the criteria for GFR for decreased HRQOL varies between studies (e.g., eGFR < 60 or < 45 mL/min/1.73 m^2^), decreased renal function is a risk factor for decreased HRQOL^11,16^. In advanced CKD, more attention is required for the deterioration of HRQOL, especially in patients with a rapid decline in kidney function.

The strength of our study was the large number of predialysis patients with CKD of all stages. The KNOW-CKD study used in the analysis is a well-designed protocol and a nationally representative cohort study. HRQOL was not cross-sectionally investigated once, but the results of follow-up results were also analyzed. In addition, this is a meaningful study because little is known about the deterioration of HRQOL due to rapid kidney function decline. However, this study has some limitations. HRQOL assessment is complex and difficult. There are geographic, cultural, linguistic, and generational variations in the HRQOL measurements. We used the Korean version 1.3 of the Kidney Disease Quality of Life short form (KDQOL-SF). In addition, the universal standard methods for assessing HRQOL are limited. We cannot rule out potential residual confounders, although we considered various confounding factors in the multivariable analysis. Finally, the study participants belonged to a single ethnic group of Koreans; thus, caution is required when generalizing our findings to other ethnicities.

In conclusion, rapid decline in kidney function was associated with rapid deterioration of HRQOL in patients with predialyis CKD. Attention should be paid to the deterioration of HRQOL in patients with a rapid decline in kidney function. Furthermore, early assessment of HRQOL deterioration in high-risk patients and attempting to modify them may help improve HRQOL in patients with CKD.

## Methods

### Participants and ethics statement

We reviewed the baseline data and follow-up results from the KNOW-CKD, a nationwide multicenter prospective cohort study that includes predialysis patients with CKD stages 1–5. The detailed study design and methods of the KNOW-CKD have been described previously^17^. Of the patients enrolled in the KNOW-CKD cohort, 970 participants had an eGFR of more than three times during the follow-up period and underwent HRQOL evaluation at enrollment and at 5-year follow-up. The study protocol was approved in 2011 by the ethical committee of each participating clinical center and by the institutional review boards of Seoul National University Hospital (1104-089-359), Yonsei University Severance Hospital (4-2011-0163), Seoul St. Mary’s Hospital (KC11OIMI0441), Seoul National University Bundang Hospital (B-1106/129-008), Kangbuk Samsung Medical Center (2011-01-076), Gil Hospital (GIRBA2553), Eulji General Hospital (201105-01), Chonnam National University Hospital (CNUH-2011-092), and Pusan Paik Hospital (11–091). This study was performed in accordance with the principles of the Declaration of Helsinki, and all study participants provided written informed consent.

### Clinical data collection and variables

Data on baseline demographic characteristics, such as age, sex, comorbidities, smoking status, and laboratory parameters at enrollment, were extracted from an electronic data management system (http://www.phactax.org) with the help of the Division of Data Management at the Seoul National University Medical Research Collaborating Center. Patients with DM were defined as those with a history of DM, those with a fasting serum glucose level of ≥ 126 mg/dL, or those on anti-diabetic medication. Patients with HTN were defined as those with a history of HTN, those with a systolic blood pressure of ≥ 140 mmHg or a diastolic blood pressure of ≥ 90 mmHg, or those on antihypertensive drugs. The following laboratory values were measured using a ≥ 8-h fasting blood sample at each participating center: hemoglobin, uric acid, albumin, total cholesterol, and C-reactive protein levels. Serum creatinine level was measured at a central laboratory (Lab Genomics, Korea) using an isotope dilution mass spectrometry-traceable method^18^. eGFR was calculated using the Chronic Kidney Disease Epidemiology Collaboration (CKD-EPI) creatinine equation^19^. CKD stages were defined according to the Kidney Disease: Improving Global Outcomes guidelines^20^. Second-voided or random urine samples were immediately sent to a central laboratory to measure urine creatinine and protein levels. Urinary protein excretion was quantified using the random urine protein-to-creatinine ratio (g/g). Low-income status was defined as a monthly family income of less than ₩1,500,000 (approximately 1500 US dollars). A low education level was defined as an academic background lower than high school graduation because high school education is compulsory in Korea. The rate of kidney function decline per year was determined using the slope of the eGFR, which was calculated using a generalized linear mixed model with a random slope and random intercept. The random slope model assumes an explanatory variable (repeated serum creatinine measurements) to be a different effect for each group with a different eGFR slope, which allows each group line to have a different slope. A random intercept model estimates a separate intercept for each individual in each group, but when the slope for each group is 0, the intercept for each individual in each group is 0, making an intercept of 0 in the entire group^21,22^. In our study protocol, all CKD patients are required to have their serum creatinine levels measured every 6 months for 1 year after enrollment and every 1 year from the first year onward. In the mixed model, we used the individual's eGFR measured at least three times as the outcome variable and an individual and repeated serum creatinine measurements as an explanatory variable. The rapid decline in kidney function was defined as a decline in eGFR of > 3 mL/min/1.73 m^2^/year. Patients were categorized into non-rapid and rapid kidney function decline groups.

### HRQOL

HRQOL was evaluated using the Korean version 1.3 of the KDQOL-SF^23,24^. The KDQOL-SF contains the Medical Outcome Study Short Form-36 Health Survey (SF-36), which measures eight domains of HRQOL comprising a PCS and an MCS. The simple SF-36 questionnaire used in this study are presented in Table S1. The PCS and MCS contain four subscales. The PCS components are physical function, role-physical limitations due to physical problems, bodily pain, and general health. Meanwhile, the MCS components are vitality, role-emotional limitations due to mental problems, social function, and mental health. Patients’ responses to the survey were translated into SF-36 scores, and each scale was scored from 0 to 100; the higher the score, the better the HRQOL of the patient. Rapid deterioration of HRQOL was defined as changes in HRQOL values higher than the median. Changes in HRQOL values were calculated as follows: (100 × [baseline HRQOL − 5-year HRQOL]/baseline HRQOL).

### Statistical analyses

Continuous variables are presented as means ± standard deviations or as medians (interquartile ranges) and were compared using the *t*-test or Mann–Whitney test. Categorical variables are presented as frequencies and percentages and were analyzed using the Chi-square test or Fisher’s exact test, as appropriate. A log transformation was used to normalize variables with a skewed distribution. We conducted PSM to minimize differences in baseline kidney function between the two groups. The propensity scores were calculated from the logistic regression models and denoted the probability of being allocated to either the non-rapid or rapid kidney function decline group. The following covariates were used to perform the logistic regression: age, sex, and eGFR. Patients were matched using the nearest neighbor method with no replacement, a 0.2 caliper width, and 1:1 matching. The non-rapid and rapid kidney function decline groups after PSM showed similar distributions of propensity scores, indicating that the differences in covariates between the two groups were minimal. The characteristics of the two groups were compared before and after PSM.

Multivariable binary logistic regression analysis was performed after adjusting for multiple confounders to identify factors related to the rapid deterioration of HRQOL. The outcomes of patients with a rapid decline in kidney function were analyzed. In the subgroup analysis using a fully adjusted multivariable binary logistic regression model, we categorized patients according to their age, sex, presence of DM, and eGFR. Statistical significance was set at *P* < 0.05. SPSS statistical software (version 20.0, IBM Co., Armonk, NY, USA) was used for all descriptive and outcome analyses, and R software version 4.1.3 (R Core Team, R Foundation for Statistical Computing, Vienna, Austria, https://www.R-project.org) was used for PSM.

## Supplementary Information

Supplementary Information.

## Data Availability

All data generated or analyzed during this study are included in this published article, supplementary data.
