# Supplementary material for: Rapid decline in kidney function is associated with rapid deterioration of health-related quality of life in chronic kidney disease
Source: Sci Rep. 2023 Jan 31;13:1786. doi: 10.1038/s41598-023-28150-w (PMC9889397; doi:10.1038/s41598-023-28150-w)
Supplement: Supplementary file 1 — Supplementary Information. [file 41598_2023_28150_MOESM1_ESM.docx]

**Supplemental Materials**

**Figure S1. Subgroup analysis for the association between rapid kidney function decline and HRQOL deterioration using a multivariable binary logistic regression model**


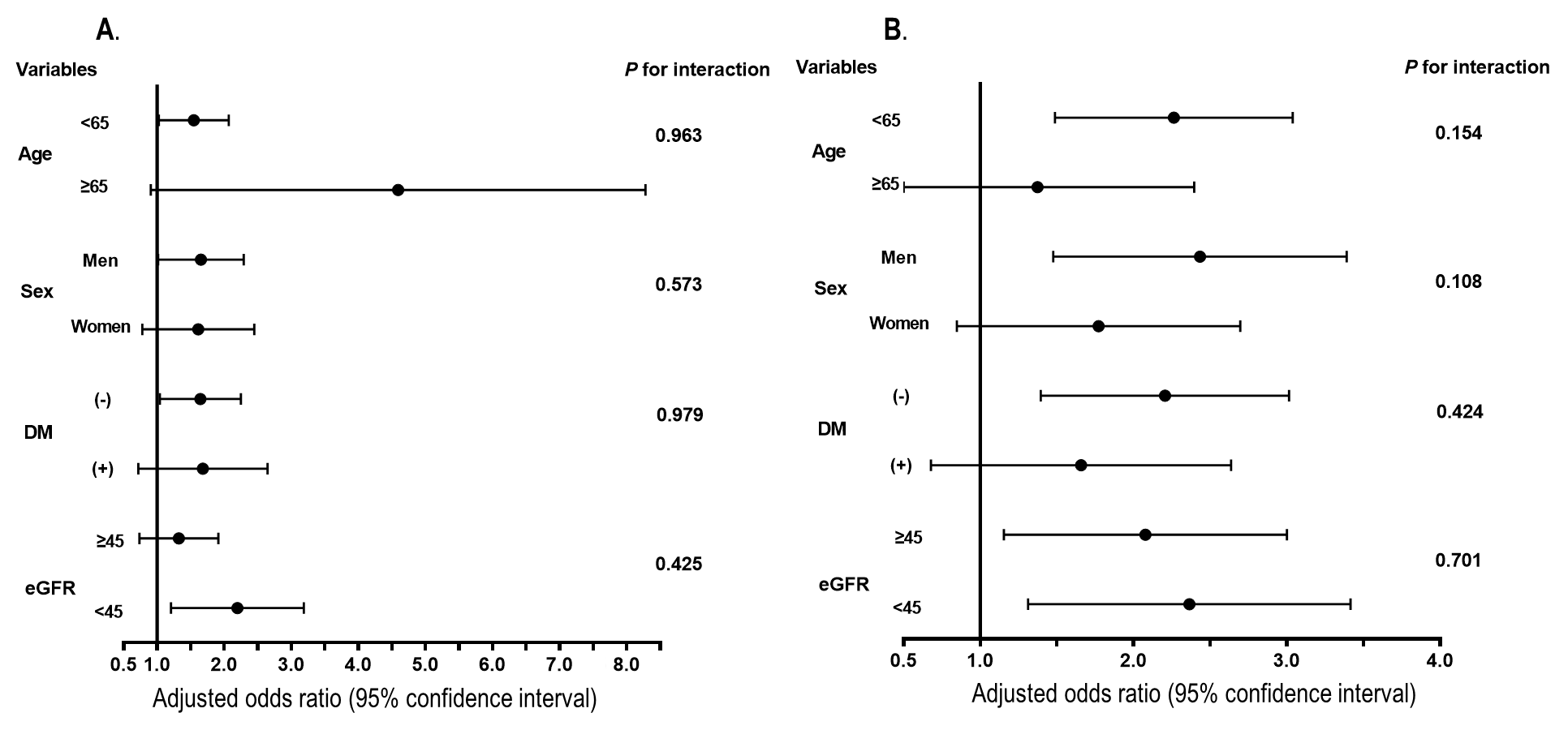

Odds ratio (OR) by subgroups adjusted for age, sex, HTN, DM, mean blood pressure, BMI, education, income, employment, hemoglobin, uric acid, albumin, and total cholesterol levels, baseline eGFR, and logUPCR

A. ORs for the deterioration of the PCS score. A rapid decline in kidney function was a significant risk factor for the rapid deterioration of the PCS score in patients with an eGFR of < 45 mL/min/1.73 m^2^, those with an age of < 65 years, or male individuals.

B. ORs for the deterioration of the MCS score. A rapid decline in kidney function was a significant risk factor for the rapid deterioration of the MCS score in patients with both an eGFR of ≥ 45 and < 45 mL/min/1.73m^2^, those with an age of < 65 years, male individuals, or patients without diabetes.

HRQOL, health-related quality of life; OR, odds ratio; PCS, physical component summary; MCS, mental component summary; DM, diabetes mellitus; HTN, hypertension; BMI, body mass index; eGFR, estimated glomerular filtration rate; UPCR, urine protein-to-creatinine ratio

**Table S1. Questionnaires of the Medical Outcome Study Short Form-36 Health Survey**

|  |  | Questionnaires of Short Form-36 Health Survey |
| --- | --- | --- |
| 1. |  | In general, would you say your health is: excellent, very good, good, fair, poor |
| 2. |  | Compared to one year ago, how would you rate your health in general now? |
| 3. |  | The following items are about activities you might do during a typical day. Does your health now limit you in these activities? If so, how much? |
|  | a | Vigorous activities, such as running, lifting heavy objects, participating in strenuous  sports |
|  | b | Moderate activities, such as moving a table, pushing a vacuum cleaner, bowling, or  playing golf |
|  | c | Lifting or carrying groceries |
|  | d | Climbing two floors of stairs |
|  | e | Climbing the half floor of stairs |
|  | f | Bending, kneeling, or stooping |
|  | g | Walking more than 1.6 kilometers |
|  | h | Walking about 200 meters |
|  | i | Walking about 50 meters |
|  | j | Bathing or dressing yourself |
| 4. |  | During the past 4 weeks, have you had any of the following problems with your work or other regular daily activities as a result of your physical health? |
|  | a | Cut down the amount of time you spent on work or other activities |
|  | b | Accomplished less than you would like |
|  | c | Were limited in the kind of work or other activities |
|  | d | Had difficulty performing the work or other activities (for example, it took extra effort) |
| 5. |  | During the past 4 weeks, have you had any of the following problems with your work or other regular daily activities as a result of any emotional problems (such as feeling depressed or anxious)? |
|  | a | Cut down the amount of time you spent on work or other activities |
|  | b | Accomplished less than you would like |
|  | c | Didn’t do work or other activities as carefully as usual |
| 6. |  | During the past 4 weeks, to what extent has your physical health or emotional problems interfered with your normal social activities with family, friends, neighbors, or groups? |
| 7. |  | How much bodily pain have you had during the past 4 weeks? |
| 8. |  | During the past 4 weeks, how much did pain interfere with your normal work (including both work outside the home and housework)? |
| 9. |  | These questions are about how you feel and how things have been with you during the past 4 weeks. |
|  | a | Did you feel full of pep? |
|  | b | Have you been a very nervous person? |
|  | c | Have you felt so down in the dumps that nothing could cheer you up? |
|  | d | Have you felt calm and peaceful? |
|  | e | Did you have a lot of energy? |
|  | f | Have you felt downhearted and blue? |
|  | g | Did you feel worn out? |
|  | h | Have you been a happy person? |
|  | i | Did you feel tired? |
| 10. |  | During the past 4 weeks, how much of the time has your physical health or emotional problems interfered with your social activities (like visiting with friends, relatives, etc.)? |
| 11. |  | Please choose the answer that best describes how true or false each of the following statements is for you. |
|  | a | I seem to get sick a little easier than other people |
|  | b | I am as healthy as anybody I know |
|  | c | I expect my health to get worse |
|  | d | My health is excellent |
| 12. |  | How true or false is each of the following statements for you? |
|  | a | My kidney disease interferes too much with my life |
|  | b | Too much of my time is spent dealing with my kidney disease |
|  | c | I feel frustrated dealing with my kidney disease |
|  | d | I feel like a burden on my family |
| 13. |  | These questions are about how you feel and how things have been going during the past 4 weeks. |
|  | a | Did you isolate yourself from people around you? |
|  | b | Did you react slowly to things that were said or done? |
|  | c | Did you act irritable toward those around you? |
|  | d | Did you have difficulty concentrating or thinking? |
|  | e | Did you get along well with other people? |
|  | f | Did you become confused? |
| 14. |  | During the past 4 weeks, to what extent were you bothered by each of the following? |
|  | a | Soreness in your muscles? |
|  | b | Chest pain? |
|  | c | Cramps? |
|  | d | Itchy skin? |
|  | e | Dry skin? |
|  | f | Shortness of breath? |
|  | g | Faintness or dizziness? |
|  | h | Lack of appetite? |
|  | i | Washed out or drained? |
|  | j | Numbness in hands or feet? |
|  | k | Nausea or upset stomach? |
|  | l | (Hemodialysis patient only) Problems with your access site? |
|  | m | (Peritoneal dialysis patient only) Problems with your catheter site? |
| 15. |  | Some people are bothered by the effects of kidney disease on their daily life, while others are not. How much does kidney disease bother you in each of the following areas? |
|  | a | Fluid restriction? |
|  | b | Dietary restriction? |
|  | c | Your ability to work around the house? |
|  | d | Your ability to travel? |
|  | e | Being dependent on doctors and other medical staff? |
|  | f | Stress or worries caused by kidney disease? |
|  | g | Your sex life? |
|  | h | Your personal appearance? |
| 16. |  | Have you had any sexual activity in the past 4 weeks?  How much of a problem was each of the following in the past 4 weeks? |
|  | a | Enjoying sex? |
|  | b | Becoming sexually aroused? |
| 17. |  | For the following question, please rate your sleep using a scale ranging from 0 representing “very bad” to 10 representing “very good.” |
| 18. |  | How often during the past 4 weeks did you |
|  | a | Awaken during the night and have trouble falling asleep again? |
|  | b | Get the amount of sleep you need? |
|  | c | Have trouble staying awake during the day? |
| 19. |  | Concerning your family and friends, how satisfied are you with |
|  | a | The amount of time you are able to spend with your family and  friends? |
|  | b | The support you receive from your family and friends? |
| 20. |  | During the past 4 weeks, did you work at a paying job? |
| 21. |  | Does your health keep you from working at a paying job? |
| 22. |  | Overall, how would you rate your health? |
| 23. |  | Think about the care you receive for kidney dialysis. In terms of your satisfaction, how would you rate the friendliness and interest shown in you as a person? |
| 24. |  | How true or false is each of the following statements? |
|  | a | Doctors and nurses encourage me to be as independent as possible |
|  | b | Doctors and nurses support me in coping with my kidney disease |
